# Acute Encephalopathic Presentation of 3-Methylglutaconic Aciduria Type I With a Novel Mutation in AUH Gene

**DOI:** 10.7759/cureus.11951

**Published:** 2020-12-07

**Authors:** Sai Chandar Dudipala, Prashanthi M, Krishna Chaithanya B, Laxman Kumar Chenalla

**Affiliations:** 1 Pediatric Neurology, Star Women and Children Hospital, Karimnagar, IND; 2 Pediatrics, Prathima Institute of Medical Sciences, Karimnagar, IND; 3 Pediatrics, Star Women and Children Hospital, Karimnagar, IND

**Keywords:** auh gene, 3-methylglutaconyl-coa hydratase, low leucine diet

## Abstract

3-Methylglutaconic aciduria type I (3-MGA I) is a rare inherited disorder of the leucine metabolism pathway due to mutations in the AUH gene for 3-methylglutaconyl-CoA hydratase enzyme and enzyme deficiency. It has a variable phenotypic presentation from infancy to adulthood. Here, we report a three-year-old female patient with normal development presented with acute encephalopathy and status dystonicus. Neuroimaging was normal. Urine organic acid analysis showed high levels of 3-methylglutaconic acid, 3-hydroxyisovaleric acid. Next-generation sequencing revealed a novel homozygous mutation of variant c.505+1G>C (5' splice site) in intron 4 of the AUH gene that was compatible with the diagnosis of 3-MGA I. The child was asymptomatic on follow-up with a low leucine diet. Clinicians should suspect rare inherited metabolic disorders in acute onset unexplainable neurological symptoms and evaluate with urine organic acid analysis.

## Introduction

3-Methylglutanoic aciduria type I (3-MGA I) is a rare autosomal recessive disorder of leucine metabolism [1]. It is caused by a deficiency of 3-methylglutaconyl-CoA hydratase (3-MGH) and characterized by markedly increased urinary excretion of 3-methylglutaconic acid (3-MGA) and mildly elevated urinary 3-methylglutaric acid (3-MG) and 3-hydroxyisovaleric acid (3-HIVA) [2, 3]. 3-MGH is encoded by the AUH gene. Clinical characteristics of 3-MGA I are heterogeneous with a wide spectrum from asymptomatic to mild neurological impairment to severe encephalopathy, psychomotor retardation, and extrapyramidal symptoms [4]. The diagnosis is usually made by urinary organic acid analysis and confirmation by AUH gene mutation analysis. So far, no specific effective treatment has been reported, but low leucine or protein diet is advised. In this study, we reported a case of 3-MGA I in a three-year-old female child presented with status dystonicus and acute encephalopathy.

## Case presentation

A three-year-old female patient presented to the emergency room with status dystonicus since morning. The child was stabilized with oxygen, intravenous midazolam. She had a fever, irritability and unable to sleep for 24 hours before admission. There was no history of trauma and toxin ingestion. She was the only child of consanguineous parents, born at term via Cesarean section with a birth weight of 3 kg. She had no significant perinatal history. The family history didn’t reveal any similar symptoms. The patient attained all her milestones, appropriate for age.

On examination, the child was irritable, pulse rate 120/min, blood pressure 90/46 mmHg, temperature 98.6° F and oxygen saturation of 96%. Neurological examination revealed irritability, central hypotonia, and dystonia with a head circumference of 48 cm. There was no other significant abnormal finding in physical and other systemic examinations.

On laboratory evaluation, liver function tests, creatinine, hemogram, serum electrolytes, calcium, magnesium, ammonia, serum lactate, creatinine phosphokinase, arterial blood gases, and C-reactive protein were normal. Dengue serology and malaria parasite were non-reactive. The electroencephalogram showed encephalopathic features. Magnetic resonance imaging (MRI) brain with contrast was normal. The cerebrospinal fluid analysis showed five cells with lymphocytic predominance, normal glucose, protein, and lactate levels. Urine organic acid analysis by gas chromatography-mass spectrometry (GCMS) revealed a high level of 3-MGA, 3-MG, and 3-HIVA. Based on the clinical and laboratory findings, 3-MGA I was considered in this patient. DNA sequencing with a next-generation sequencing platform revealed a pathogenic mutation AUH gene. Previously, the mutation has been reported in literature rarely. Family screening and genetic counseling were advised.

The child was treated symptomatically with clonazepam, trihexyphenidyl, proper hydration, intermittent midazolam. The child improved gradually and was discharged on the seventh day of admission. Parents were counseled about the condition and advised a low leucine diet. 

On follow-up, the child became normal and slowly stopped trihexyphenidyl and continued on a low leucine diet. At present, after two years of illness, the child was normal, and no neurological abnormality was noticed.

## Discussion

3-MGA-I is a rare inherited metabolic disorder. Since 1976, only about 40 cases have been reported. The true incidence of this condition may be underestimated because some patients are asymptomatic, and there is a lack of neonatal screening programs [4-8]. It is a clinically heterogeneous group of neurological disorders that is characterized by a wide clinical spectrum, including asymptomatic patients, delayed speech development, severe encephalopathy, psychomotor retardation, and extrapyramidal symptoms [4]. In this case, the child was neurologically normal but presented with unexplained encephalopathy with status dystonicus during fever. A similar case was reported by Gibson et al. [9]. Narisawa et al. reported a two-year-old child with motor and speech delay and unexplained encephalopathy [3].

In children with unexplained encephalopathy, urine organic acid analysis aids in the diagnosis. In this case, after a routine metabolic panel, GCMS analysis was done. GCMS analysis revealed a high level of 3-MGA, 3-MG, and 3-HIVA, which was consistent with 3-MGA I. The diagnosis helps to restrict leucine in diet and improvement in symptoms.

MRI brain findings of 3-MGA I are nonspecific, including normal, basal ganglia lesions, nonspecific white matter lesions [6]. In our case report, the MRI brain was normal.

The gene for 3-MGH enzyme AUH is mapped on chromosome 9q22.3. Up to now, 11 different types of mutations in the AUH gene have been reported [7, 8]. In the present case, DNA analysis with next-generation sequencing showed a novel mutation of variant c.505+1G>C (5' splice site) in intron 4 of the AUH gene in a homozygous state. In literature, rare case series have been reported with this gene mutation [7, 8, 10, 11]. Ly et al. reported a variety of AUH gene mutations in five patients from four independent families [10]. It is also possible that the pathogenesis of AUH deficiency is related to metabolic stress like infection, fever, or fasting. In the present case, the child became symptomatic during fever. However, this has not been consistently observed in known patients with 3-MGA I.

At present, there are no specific therapeutic options available for the treatment of 3 MGA I. However, leucine restricted diet will help to improve neurological outcomes [11]. In the present case, the child was improved, and there were no other neurological abnormalities with a low leucine diet.

## Conclusions

The purpose of this report is to keep attention to rare metabolic disorders that have no previous neurological illness and present with the atypical unexplained presentation. In such cases, metabolic evaluation with GCMS will be helpful, and early diet restriction could help improve the neurological state or prevent acute illness during stress, as in this case report.
